# An AI-Enabled Predictive Harm Response Management Algorithmic Tool to Reduce Adverse Events in Health Care Settings (PreHaRM): Protocol for a Three-Phase Model Development and Pilot-Testing Study

**DOI:** 10.2196/75474

**Published:** 2026-05-27

**Authors:** Marion Eckert, Greg Sharplin, Wolfgang Mayer, Georg Grossmann, Jan Stanek, Gourav Gupta, Lachlan Darch, Markus Stumptner, Nicholas Marlow

**Affiliations:** 1 Rosemary Bryant AO Research Centre, College of Health, Adelaide University Adelaide, South Australia Australia; 2 Industrial AI Research Centre School of Computer Science and Information Technology, Adelaide University Adelaide, South Australia Australia

**Keywords:** nursing, midwifery, artificial intelligence, AI, risk prediction, patient falls, medication error, violence and aggression, code black

## Abstract

**Background:**

Current data analysis and coordination methods do not effectively support nurses and midwives in risk reduction, as retrospective reporting does not allow real-time insights and precludes proactive, preventive care. Analysis of administrative data within Australia’s health care sector to predict risk may help address this shortcoming. Predictive analytics can transform these data into meaningful insights, identifying harm risk profiles that benefit the performance of Australian and international clinical programs. Importantly, these tools may support nurses and midwives in preventing adverse events and predicting high-risk situations. Researchers, in collaboration with local health network staff, will develop a proof-of-concept predictive risk algorithm. The “predictive harm response management algorithmic tool to reduce adverse events in healthcare settings” program (project DHCRC-0156) will provide real-time insights via an interactive dashboard, enabling nurses, midwives, and health administration to assess risks and optimize resources in health care settings. This protocol details the algorithm development activities for subproject 1a, predictive risk model development, which aims to develop and pilot-test a predictive harm algorithm across 2 South Australian local health networks.

**Objective:**

This study aims to identify the clinical harm outcome of interest and relevant data sources per site and build a suitable data solution to model predictors of harm risk and identify actionable clinical, workforce, and environmental factors that affect the harm outcome of interest.

**Methods:**

This study design includes three phases: (1) model generation, (2) model evaluation, and (3) prototype development. Data linkage by SA-NT DataLink can only proceed following approval from each of the following: the South Australia Department for Health and Wellbeing Human Research Ethics Committee, the University of South Australia Human Research Ethics Committee, and hospital governance committees. The clinical dataset will be split into a training set, a validation set, and a test set. Exploratory data analysis will be undertaken to ascertain features and classify outcomes from the raw dataset. Derived features will be computed, feature correlations will be estimated, and initial feature selection will be performed. Iterative model development will occur over 3 stages, and a dashboard to display these results will be developed.

**Results:**

The study commenced on July 19, 2021, and will conclude on December 31, 2025. Finalized results are expected in December 2025.

**Conclusions:**

This research will conclude with the development of the algorithm for transferability to health care environments. Research activities will be detailed in publicly available reports and manuscripts prepared for peer-reviewed journals that will be drafted in accordance with existing and appropriate checklists.

**International Registered Report Identifier (IRRID):**

DERR1-10.2196/75474

## Introduction

Adverse events occur despite the efforts and diligence of health professionals. Their occurrence can have a significant impact on patients, families, nursing and midwifery staff, and the health care system as a whole. Across the Australian health care system, patient falls, medication errors, and instances of violence and aggression (code black) have a frequency and consequence that are as pronounced as they are avoidable.

Hospital falls represent a significant challenge in health care settings. A multisite prospective cohort study conducted from 2011 to 2013 across 12 acute medical and surgical wards in 6 Australian hospitals found that, among 27,026 hospital admissions, 3.6% involved at least one fall and 1.2% resulted in a fall-related injury [1]. Estimates suggest that the cost per fall in a health care setting ranges from approximately Aus $10,000 (US $7000) to Aus $40,000 (US $30,000) [1,2].

Following falls, medication errors are the second most frequently reported incidents in health care settings [3] and are defined as mistakes occurring at any stage of the medication process, whether ordering, dispensing, administering, or monitoring, that result in harm or pose a risk to patients [4]. It is estimated that medication error–related hospitalization in Australia costs approximately Aus $1.2 billion (US $840 million) annually [5].

Although all health care professionals are at risk, nurses and midwives often face the highest risk of assault, with research estimating that approximately 67% of nurses are likely to have experienced some kind of violence and aggression in the previous year [6,7]. A systematic review identified that overall exposure to violence toward nurses can be estimated at 36.4% for physical violence, 66.9% for nonphysical violence, 39.7% for bullying, and 25% for sexual harassment [8]. Beyond the direct distress and physical harm caused by such violence, there is a range of adverse outcomes, including increased risk of psychological disorders such as posttraumatic stress disorder and depression [9,10] and higher rates of occupational burnout and absenteeism [11,12].

Ward workforce availability and composition can affect patient outcomes, with lower skill mix levels linked to longer stays and reduced nurse and midwife numbers leading to an increase in adverse patient outcomes, including “mortality, infections, falls, and longer lengths of stay” [13]. Skilled care has substantial potential to prevent hospital-acquired complications as nurses and midwives spend the most time at the bedside and directly influence patient outcomes. Determining the optimal balance of trained professionals is essential to maintaining high-quality care while managing staffing costs.

The vast amount of routine administrative data collected and stored within the Australian health care sector holds a wealth of insightful and actionable information. Accessing and analyzing these data could enable improvements in clinical processes, enhance patient safety and outcomes, and support future workforce planning. Although a significant amount of information is gathered, routine data are not being used to their full potential. Exploring different approaches is necessary given the substantial impact that these events have on the health budget and individuals. Currently, data analysis and reporting methods do not effectively assist nurses and midwives in risk reduction as existing retrospective reports fail to provide real-time risk insights, thereby limiting proactive care. Predictive analytics can leverage these data to offer meaningful insights into the harm risk profiles of Australian and international clinical programs, helping nursing and midwifery staff proactively minimize the likelihood of adverse events and forecast the emergence of high-risk clinical situations.

An initial pilot of hospital wards and workforce data by the Rosemary Bryant AO Research Centre at the University of South Australia (UniSA) in collaboration with Central Adelaide Local Health Network (CALHN) identified factors that contributed to adverse events. Interest in adverse event prevention was also confirmed by the Southern Adelaide Local Health Network (SALHN). On the basis of this need and mutual interest, a proposal to develop a prognostic predictive risk algorithm was submitted to the Digital Health Cooperative Research Centre. The resultant “PreHaRM: predictive harm response management algorithmic tool to reduce adverse events in healthcare settings” program (PreHaRM project) was funded to develop a predictive risk tool (PreHaRM tool) for use within the CALHN and SALHN, with a view to being delivered across other networks. Development of the PreHaRM tool has proceeded in collaboration with the Industrial AI Research Centre at UniSA, SA-NT DataLink, the local health networks (LHNs), and SA Health. This research is currently building the PreHaRM tool as a visual, interactive application for ward nurses and midwives, unit managers, and health administrators to view real-time insights, input variables, and data of their own and produce assessments and resource predictions based on real clinical and administrative data.

The PreHaRM project is a multistage program of work. This protocol focuses solely on the methodology for developing the PreHaRM algorithm (subproject 1a: predictive risk model development). Additional protocols detailing user interface design methods have been published [14], with a further implementation protocol under development.

## Methods

The aim is to develop, pilot-test, and implement predictive harm algorithms for South Australia’s 2 largest health networks: CALHN and SALHN. The aim of this PreHaRM project is to identify the clinical harm outcomes of interest (medication errors, violence and aggression, and falls) and relevant data sources at each site and create an effective data solution to model the predictors of harm risk for each outcome.

### Design

Scoping activities preceded model development to assess platform requirements and the ability to access necessary datasets. The need was identified through targeted discussions with senior staff from the LHN, who outlined the adverse events of interest. This was followed by engagement with digital health leads at the LHNs of interest (CALHN and SALHN) to verify the available datasets and the relevant date ranges accessible. After completing these activities, data approvals, access, deidentification, linkage, and receipt of datasets proceeded, with algorithm development starting in November 2024. The project is now in the model generation phase and will soon move to the model evaluation phase. The UniSA Industrial AI Research Centre will oversee both phases.

This research examined health service activity data collected between July 1 2019 and June 30, 2022 from the following locations:

- CALHN—Royal Adelaide Hospital and Queen Elizabeth Hospital: inpatient and general medical wards
- SALHN—Flinders Medical Centre Emergency Department: all general medical wards and the Margaret Tobin Centre

The data were enriched with initial feature selection and feature engineering using domain knowledge to identify potentially useful intrinsic and time-varying features. Categorical features were encoded as one-hot vectors. Patient, staff, and ward variables were further processed to derive aggregate features capturing historical evolution. Features aggregating relevant variables over the preceding 12 weeks and over the entire prior time span were computed for each time point of interest (the shift start and the incident time). Aggregate features captured the mean and the sum of numeric features within each window. For multivalued categorical features, the entropy of each feature was computed for each window. Overall, 844 features and approximately 1.79 million records at the shift level of granularity were used to develop and test the models.

The data were partitioned into training (80%), validation (10%), and test (10%) sets considering the imbalance in the target variable and the temporal changes in features. The split was based on periods to avoid information leakage into the validation and test datasets, starting with the initial time span for the training set; then the validation set; and, finally, the test set. This method follows standard practices for time-series forecasting and is appropriate for situations in which data accumulate over time.

Feature correlation analysis and feature selection were conducted on the training dataset before model training. Highly correlated features were identified using the φ*_k_* correlation coefficients [15] (threshold of 0.5), and one feature was selected from each correlated group. Additionally, the importance of the remaining features was assessed using a random forest feature importance estimator, keeping only those exceeding the median importance score.

During the model generation phase, individual predictive classification models were trained to predict each adverse event of interest. A suitable model architecture was selected to account for the imbalance in the target variable and patient cohort distributions, the temporal variation in features, and the high dimensionality of the dataset. Classification model architectures, such as weighted logistic regression, random forest, gradient boosting, and recurrent neural networks (RNNs), were considered. Logistic regression, random forest, and neural networks are well-established methods that can handle classification tasks with both categorical and continuous inputs. Weighted logistic regression offers a reliable baseline that can directly address imbalance by assigning class weights. Random forest can also be configured to balance class weights. RNNs are particularly effective at capturing the temporal features of data. Hybrid model architectures, which combine various types of models, are common in prediction solutions that accept diverse inputs. Class weighting, undersampling the majority class, and gradient boosting are complementary strategies for addressing imbalance in the outcome. As of November 2025, and in accordance with the task outlines mentioned above, a variety of models have been applied, with random forest providing the best results in terms of accuracy and precision on specific subsets of the dataset so far. Thorough validation and optimization of the RNN model remain to be completed before selecting the final model. The models were developed using a Python development framework (Python Software Foundation) and the scikit-learn (Google Summer of Code project) and PyTorch (Meta AI) libraries.

In a subsequent model evaluation phase, models will be assessed using metrics including specificity, sensitivity, *F*_1_-score, receiver operating characteristic curve, and validation set accuracy. The experiment will be repeated multiple times to minimize the effects of stochastic variation. The best model will be chosen based on an acceptable precision-recall (receiver operating characteristic curve) trade-off as determined by clinical experts.

The final chosen models will be evaluated on the independent test set to obtain an unbiased estimation of predictive outcomes. The results of this and all preceding phases will be reported using the TRIPOD-AI (Transparent Reporting of a Multivariable Prediction Model for Individual Prognosis or Diagnosis–Artificial Intelligence) guidelines [16].

The contribution of specific features to predicted risk outcomes will be quantified using statistical and feature importance analysis techniques. Shapley additive explanations [17] will be used to estimate the importance of individual features, and these importance scores will be aggregated to determine the importance of patient-, ward-, and staffing-related feature categories.

### Ethical Considerations

All research data have been and will be stored on secure servers within the UniSA information and communications technology system and accessible only to researchers. Project information is expected to be retained for a maximum of 5 years after the publication of the findings in accordance with the National Health and Medical Research Council guidelines for research data [18]. Written approval will be obtained from the Dean of Research (UniSA Clinical and Health Sciences) to delete all data files once the retention period has passed, accompanied by a digital data destruction certificate confirming that the university has securely destroyed the data.

Participant consent was not sought as data provided to researchers were deidentified by SA-NT DataLink. The method and restrictions surrounding this process was approved by the South Australia Department for Health and Wellbeing Human Research Ethics Committee, the University of South Australia Human Research Ethics Committee, and hospital governance committees. This project has sought and received each of the following ethics approvals:

- SA Health Department for Health and Wellbeing (DHW) ethics approval (2022/HRE00131)
- UniSA ethics approval (204143)
- SA Health DHW governance approval (2022/SSA00288)
- Royal Adelaide Hospital governance approval (2022/SSA00273)
- Queen Elizabeth Hospital governance approval (2022/SSA00274)
- Flinders Medical Centre governance approval (2022/SSA00275)
- Population Health Research Network approval (P0081_2021)

Oversight of project activities will be performed by a project control group that includes executive representation from each of the stakeholders: UniSA, CALHN, SALHN, the SA Health DHW, SA-NT DataLink, and the Digital Health Cooperative Research Centre. In addition, 3 working groups have been established: a technical architecture group that discusses details related to the development of the algorithm and the development of the software solution and 2 clinical implementation groups, one per LHN, that discuss dataset-related details, LHN implementation, and current issues within the health system that may affect project delivery.

## Results

The study commenced on June 21, 2021, and will conclude on December 31, 2025. Data were transferred from CALHN and SALHN hospitals to SA-NT DataLink, which deidentified and linked patient data for release to the UniSA computing facility. This process was approved through ethics agreements and governed by CALHN and SALHN governance authorizations. These administrative approvals ensured that due process for data handling was followed throughout the transfer. The facility received the final dataset in November 2024. Upon receipt, data stewardship was managed by the UniSA Industrial AI Research Centre.

Data were obtained from records already collected during clinical practice. These records included clinical admission data (71 variables), emergency department presentation data (46 variables), safety management system records (14 variables), roster data (21 variables), staff information (29 variables), and community mental health records (61 variables). Due to the comprehensive nature of the setting, describing each variable in this paper is impractical. All available variables were included in the dataset except for a free-text comment field in the roster data. The records, received in deidentified form, were linked using provided linkage keys and/or temporal alignment to create a comprehensive dataset.

Records that did not meet the basic inclusion criteria (ward, period, and event of interest) and records that could not be reliably deidentified were excluded. This study relied on deidentified patient and clinical records linked via an intermediary (SA-NT DataLink); however, due to ethics concerns regarding anonymity, data from wards with fewer than 10 patients were excluded. Moreover, records with essential fields missing and those that could be linked unambiguously were excluded.

An exploratory data analysis was conducted to gain an initial understanding of the datasets and evaluate their suitability for model development. Drawing on medical and organizational expertise, the relevance of the data was confirmed qualitatively. This process took place between November 2024 and April 2025

Final results are expected in April 2026. Anticipated outcomes include the PreHaRM tool, consisting of the algorithm and dashboard designed to identify and display the likelihood of adverse events to support nurse decision-making, as well as guidance for local staff on how to implement the tool within their LHN. It is planned that, where suitable, research activities will be detailed in publicly accessible reports and manuscripts for peer-reviewed journals. When relevant, reporting will follow the TRIPOD-AI checklist [16]. The project, along with any available results, will also be presented at scientific and public conferences, noting that no individual-level or identifiable data will be reported.

## Discussion

The anticipated outcome of this study is a functional artificial intelligence (AI)–enabled prognostic risk tool. The PreHaRM tool will include models capable of predicting the likelihood of each of the 3 adverse events of interest. These models will be integrated into a dashboard that presents the probability of each adverse event for individual patients, enabling nursing staff to intervene in a timely manner. Locally, AI-powered clinical decision support tools are gaining traction, as demonstrated by the LHNs involved in developing the Adelaide Score. This system targets general surgery patients to assess their “readiness for discharge” [19]. Their work highlights regional interest in AI-driven efficiencies among local government and health networks. While the Adelaide Score focuses on ensuring a smooth patient journey, the PreHaRM tool aims to forecast patient adversity.

Potential limitations of this research include the need for consistent data to produce meaningful forecasts. Although not unique to this project, the model’s ability to operate effectively in other settings with similar datasets has not yet been assessed but remains of interest to researchers. Similarly, the model uses static data and has not yet been tested on live data feeds; its ability to work effectively when the incoming data may not be as “clean” as the training data is also a concern for researchers. It is important to note that these issues were beyond the scope of this study but will be explored in future research.

The planned implementation and adoption of the PreHaRM tool will enable nursing and midwifery staff to identify harm and reduce risks that could lead to adverse events. This resource planning, supported by PreHaRM insights, will strengthen participating LHNs when dealing with resource constraints. The implementation process will follow the staged clinical AI implementation (SALIENT) framework, starting with a “silent trial” currently scheduled for 2026 to 2027 (approvals pending) [20].

Although subject to considerable delays, this project has endured and applied the method outlined above and now approaches the final stages of bringing this research together. As adverse events continue to besiege the Australian health care system, it is hoped that the PreHaRM tool will soon be available to assist.

## Abbreviations

AI: artificial intelligence
CALHN: Central Adelaide Local Health Network
DHW: Department for Health and Wellbeing
LHN: local health network
RNN: recurrent neural network
SALHN: Southern Adelaide Local Health Network
TRIPOD-AI: Transparent Reporting of a Multivariable Prediction Model for Individual Prognosis or Diagnosis–Artificial Intelligence
UniSA: University of South Australia
